# The Predictive Role of Subcutaneous Adipose Tissue in the Pathogenesis of Obstructive Sleep Apnoea

**DOI:** 10.3390/life12101504

**Published:** 2022-09-27

**Authors:** Viktória Molnár, Zoltán Lakner, András Molnár, Dávid László Tárnoki, Ádám Domonkos Tárnoki, László Kunos, László Tamás

**Affiliations:** 1 Department of Otolaryngology and Head and Neck Surgery, Semmelweis University, 1083 Budapest, Hungary; 2 Szent István Campus, Hungarian University of Agriculture and Life Sciences, 2100 Gödöllő, Hungary; 3 Medical Imaging Centre, Semmelweis University, 1083 Budapest, Hungary; 4 Department of Pulmonology, Pulmonology Hospital of Törökbálint, 2045 Törökbálint, Hungary; 5 Department of Voice, Speech and Swallowing Therapy, Faculty of Health Sciences, Semmelweis University, 1083 Budapest, Hungary

**Keywords:** obstructive sleep apnoea, ultrasound, obesity, subcutaneous adipose tissue thickness, artificial intelligence, drug-induced sleep endoscopy

## Abstract

**Simple Summary:**

Although several methods are used to diagnose obstructive sleep apnoea (OSA), the disorder is still underdiagnosed, leading to public healthcare problems. The main aim of the present study was to analyse the role of artificial intelligence in OSA diagnostics and obstruction localisation and, moreover, the role of subcutaneous adipose tissue in OSA pathophysiology. The significance of the present investigation is that using US in OSA diagnostics and obstruction location, an additional opportunity besides standard procedures (i.e., drug-induced sleep endoscopy or polygraphy) is presented, which is vital due to the high number of undiagnosed cases. Applying the algorithm, including artificial intelligence, the presence of obstructions and its localisation, can be determined with high precision. This can be essential in therapy planning or preoperative patient preparation.

**Abstract:**

Introduction: Our aim was to investigate the applicability of artificial intelligence in predicting obstructive sleep apnoea (OSA) and upper airway obstruction using ultrasound (US) measurements of subcutaneous adipose tissues (SAT) in the regions of the neck, chest and abdomen. Methods: One hundred patients were divided into mild (32), moderately severe-severe (32) OSA and non-OSA (36), according to the results of the polysomnography. These patients were examined using anthropometric measurements and US of SAT and drug-induced sleep endoscopy. Results: Using SAT US and anthropometric parameters, oropharyngeal obstruction could be predicted in 64% and tongue-based obstruction in 72%. In predicting oropharyngeal obstruction, BMI, abdominal and hip circumferences, submental SAT and SAT above the second intercostal space on the left were identified as essential parameters. Furthermore, tongue-based obstruction was predicted mainly by height, SAT measured 2 cm above the umbilicus and submental SAT. The OSA prediction was successful in 97% using the parameters mentioned above. Moreover, other parameters, such as US-based SAT, with SAT measured 2 cm above the umbilicus and both-sided SAT above the second intercostal spaces as the most important ones. Discussion: Based on our results, several categories of OSA can be predicted using artificial intelligence with high precision by using SAT and anthropometric parameters.

## 1. Introduction

Obstructive sleep apnoea (OSA) is the most common type of sleep-related breathing disorder, characterised by obstruction of the upper airways during sleep, resulting in hypoxia, hypercapnia and fragmentation of sleep [1]. It is believed to be an essential public health problem, as unattended cases can be responsible for cardiovascular and cerebrovascular disorders [1], cognitive [2] and sexual [3] problems, as well as traffic accidents [1].

Obesity was found to be the most critical risk factor for OSA, followed by ageing, male sex and craniofacial malformations [1]. Obesity, nowadays also mentioned as a’ global pandemic’, has shown an increasing, country-specific tendency in the last three decades [4]. It is a major problem in developed countries, resulting from increased calorie intake, lack of physical activity and changes in the gut microbiome [5]. An investigation between 1980 and 2013 showed that the prevalence of overweight and obesity increased by 27.5% in adults and 41.7% in infants [6]. The increasing tendency to obesity leads to a dynamic increase in the prevalence of OSA, which can also be observed in the inflammation and dysfunction of the subcutaneous adipose tissue resulting from intermittent hypoxia. [7]. Artificial intelligence is increasingly significant in many scientific fields and medical sciences. Due to the increasing tendency of examination results and mathematical-statistical methods, it is possible to predict OSA using artificial intelligence. Due to the complexity of OSA pathophysiology, various diagnostic methods are used; therefore, the correlations between the variables are complex and may not be analysed using conventional statistical methods. For this reason, learning algorithms (e.g., deep learning methods) are especially significant. The original biostatistical models were based on simple correspondences (e.g., linear or logarithmic) between different variables; therefore, the analyses aimed to describe these correlations [8]. However, there are some theoretical and practical issues regarding these methods. In terms of the current investigation, three are crucial. First, the relatively simple functions applied can generally limitedly be used only to describe complex biological systems with circular causality [9]. Furthermore, to apply the generally used models (e.g., logistic regression), a relatively large number of variables is necessary in order to achieve reliable results. A gold standard rule of logistic regression is that the number of variables in one cell must be at least five. In the case of three stages (e.g., low, medium and high) of ten independent variables, this means 10 × 3 × 5; therefore, 150 answer givers are suspected. However, the distribution between the different stages is unequal; hence, more answers are needed for a safety model that fits at least triple or quintuple. According to Bujang et al., at least 500 variables are necessary for a clinical investigation using logistic regression [10]. Furthermore, in traditional statistical models (e.g., confirmative, multiple regression analysis), the researcher tests a correlation system based on previous scientific literature or experiences [11]. However, the model might not correctly reflect reality, as it may give away the opportunity to analyse more complex correlations [12]. It is possible to analyte the significant number of variables taken from diagnostic methods using artificial intelligence and non-linear correlations can also be analysed. The advantages of artificial intelligence can be widely used in medical studies, i.e., in diagnostics, therapy, or prediction. A study on artificial intelligence-based OSA prediction performed by a generalised regression neural network conducted a diagnosis including 23 clinical parameters [13], while another study successfully predicted with AHI [14]. Prediction of OSA was also possible including the most vital clinical factors of OSA, i.e., sex, age, BMI and snoring status, using a neural network [15].

Consequently, the correspondences between OSA and anthropometric parameters and anatomical characteristics can be analysed. By analysing these correlations, OSA pathophysiology can be studied and diagnostic methods can be expanded. Additionally, there is the possibility to determine upper airway obstruction and build prediction models. Algorithms based on artificial intelligence can improve prediction using mathematical models using a learning method on more data points. Determining the anatomical characteristics of OSA patients is possible using anthropometric measurements, sleep endoscopy and medical imaging (i.e., ultrasound or MRI). This data collection allows the construction of a cost-effective diagnostic model of OSA, including the determination of the location and severity of the obstructions.

Although polysomnography (PSG) is the diagnostic method for OSA, due to the high number of patients, the use of screening methods (e.g., polygraphy or portable home devices) was necessary.

Hence, the present study aimed to analyse the role of the basic anthropometric parameters and the results of SAT US measurements in the pathogenesis of upper airway obstruction using artificial intelligence. By building an algorithm, it is possible to help clinicians in everyday practice in decision making, applying a relatively inexpensive and easy-to-use algorithm. The US-based SAT examinations were based on US examinations with other indications (e.g., thyroid gland abnormalities or bilestones) as a complementary examination of standard US measurements.

## 2. Materials and Methods

### 2.1. Participants

**Figure 1:**
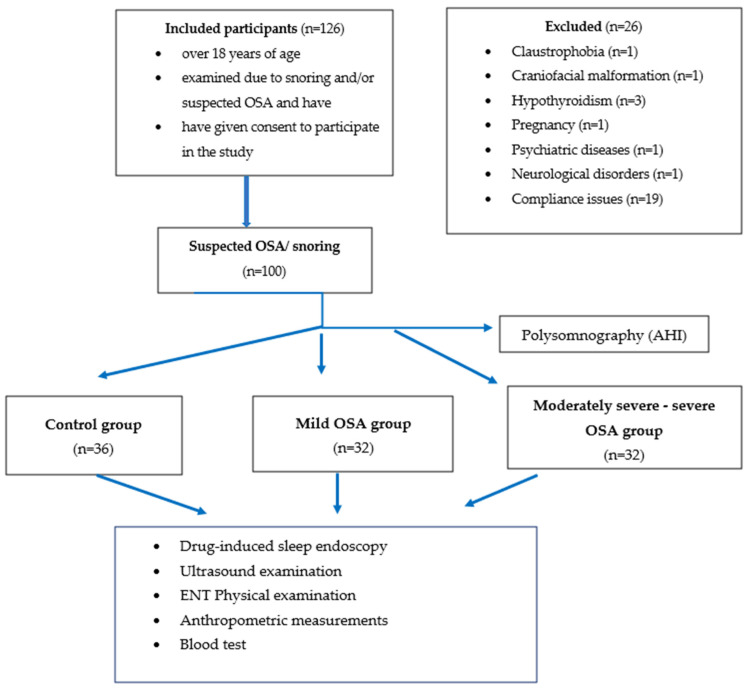
Study Population flow chart.

In this prospective investigation, one hundred patients who met the inclusion criteria (74 men and 26 women, mean age SD, 42.15 ± 11.7 years) at the Department of Otolaryngology and Head and Neck Surgery of Semmelweis University were consecutively enrolled. Detailed examination of the patients consisted of case history, physical otorhinolaryngological examinations, polysomnography and US measurements. Only patients over 18 years of age, examined due to snoring and/or suspected OSA and who have given their consent to participate in the study were included. Patients with previous otorhinolaryngological or oral surgeries, suffering from hypo- or hyperthyroidism, who had craniofacial trauma or malformation (i.e., Down syndrome), who have claustrophobia, connective tissue diseases, neurologic or psychiatric disorders, including overuse of drugs or alcohol, and pregnancy have been excluded from the study. Figure 1. shows the study flow chart.

The study was approved by the Hungarian Research Ethics Authority (National Institute of Pharmacy and Nutrition, approval reference number: 2788/2019). All patients gave their informed consent in writing.

### 2.2. Anthropometric Measurements

Basic anthropometric parameters were obtained from participants, including demographic data, gender, age, body mass, weight and height of the patients, along with the circumferences of the neck, hip and abdomen. Neck circumference was measured while the patient was standing on the cricothyroid membrane using a flexible tape measure. Hip circumference was measured at the anterior superior iliac spine, while abdominal circumference was measured at the level of the umbilicus. The BMI parameters were calculated according to the height and body mass of the patients. Based on the data, the patients were divided into normal (<25 kg/m^2^), overweight (25–30 kg/m^2^) and obese (30 kg/m^2^<) groups.

### 2.3. Sleep Test

Overnight polysomnography (PSG) was performed under medical control at the Törökbálint Institute of Pulmonology using a SOMNOscreen Plus PSG device (SOMNOmedics GmbH, Germany). The definition of apnoea and hypopnea was obtained from the recommendation of the American Academy of Sleep Medicine. Apnoea is present when 90% or more airflow reduction is detected for at least 10 s. Hypopnea is a reduction of 30% or more airflow for at least 10 s, accompanied by oxyhaemoglobin desaturation (3% or more) or arousal [16]. The severity of OSA can be calculated using the apnoea-hypopnea index (AHI), which demonstrates the apnoea and hypopnea events per hour [16]. Given the relatively low number of subjects examined, our patients were grouped as normal (AHI < 5), mild (5 ≤ AHI < 15) and moderately severe-severe (AHI ≥ 15).

### 2.4. Ultrasound Measurements

The US examinations were performed at the Medical Imaging Centre of Semmelweis University using a Samsung RS85 US device (Samsung Electronics Co., Ltd., Seoul, South Korea). All measurements were performed by the same experienced radiologist, who was not informed of the previous results of the examinations of the patients. During the measurements, the subjects were lying supine, with their head in a slightly extended position. The measurements of the submental SAT region were carried out using an L3-12A linear (3–12 MHz) transducer, in a grey scale B mode, between the mandible and the hyoid bone. The thickness of the neck SAT was also measured on both sides at the level of the carotid bifurcation over the sternocleidomastoid muscles [17]. Thoracic and abdominal SAT was measured with the same transducer. In the thoracic region, the thickness of the SAT was measured over the second and third intercostal spaces. The abdominal SAT was measured 2 cm above the umbilicus in the midline; transducers were placed at a right angle softly to the skin to avoid compression of the SAT [18]. The patients were asked to breathe normally during the examinations. All measurements were made three times and the parameters were averaged.

### 2.5. Blood Test

To analyse blood lipid profiles, Total cholesterol (mmol/L), HDL-cholesterol (mmol/L), LDL-cholesterol (mmol/L) and Triglyceride (mmol/L) levels were calculated, using a venous blood sample.

### 2.6. Drug-Induced Sleep Endoscopy

Drug-induced sleep endoscopy was performed in an operating room. During the endoscopy, the participants were lying supine. 1.5 mg/kg of propofol was applied for sedation and participants were monitored with an electrocardiogram, pulse oximeter and blood pressure test. Sleep endoscopy was performed using an Olympus endoscope with a diameter of 3.5 mm by an experienced otorhinolaryngologist. The VOTE classification was used to interpret the results, indicating V = velum, O = oropharyngeal, T = tongue base and E = epiglottis. The configuration of obstruction can be anteroposterior, lateral, or concentric. The severity of the obstruction can be X = obstruction cannot be visualised, 0 = no obstruction, 1 = partial obstruction or 2 = total obstruction. Examination allows for a real-time 3D visualisation of the upper airways [19].

### 2.7. Statistical Analysis

Statistically, our objective was relatively simple, i.e., to find a robust and reliable formula for the prognosis of obstruction location and OSA. Practically, this was more difficult, as the number of cases was relatively limited and the causes behind obstruction and OSA were complex.

In the preparatory phase of our work, a wide array of different algorithms (e.g., log-linear analysis, linear and quadratic discriminant analyses) were tested, although we were unable to achieve any results due to the high number of empty cells.

Therefore, the use of algorithms developed for chemometrics was considered. This part of statistics is particularly essential to us, as in the case of the statistical analysis of different chemical results (e.g., spectroscopic data) a general phenomenon is that the number of observations is relatively low compared to the number of variables [20]. The analysis of these ‘fat’ matrices could be a promising possibility, although the sensitivity and specificity of the generally used chemometric algorithms (e.g., flexible discriminant analysis) were also unsatisfactory [21].

Consequently, the applicability of the different neural network algorithms has been tested. Neural networks are sets of connected processors, called neurons, as metaphors for nervous systems. The input neurons will be activated as a consequence of the input information, while other neurons will be activated by a series of their weighted connections from previously activated neurons [22]. In classic cases, there are layers of neurons. In the case of deep learning, the systems use many layers of neurons, giving the possibility of using more efficient algorithms [23].

This gives a possibility of using more efficient algorithms. Due to the relative deviation of the independent variables and the complex pathophysiological network correlations between OSA and these variables, our analyses were based on deep learning methods. These methods have only been introduced in recent years, possibly due to software and hardware developments [24].

Deep learning methods use networks for classification and the weights express the relative importance of these nodes. Constrasting a regressional model, numerous weights are attached to the independent variables in a neural network system. The Garson method quantified the relative importance of an independent variable for the dependent variable (response). The importance of different independent variables is dimensionless numbers, ranging between 0 and 1 [25].

Another promising approach is the random forest classification. This approach is based on the cyclical large-scale application of decision trees [26]. In the framework of our study for the calculations, the DeepNet R software package and the Random Forest package were applied. The R language and environment was used since this software is suitable for clinical data science applications [27].

Random forest [28] and deep learning algorithms [29] are widely applied for modern analysis of biostatistical data. (The use of the conventional statistical methods is highly influenced by the investigated data (e.g., normal distribution of the parameters) and only the correlation of a limited number of previously determined parameters can be analysed.

The most accurate results were obtained by applying the Deepnet r package [30] and by the random forest method of the caret package [31]. The relative importance of the different variables was analysed by the NeuralNetTools R package [32].

The normality of the parameters was tested using the Kolmogorov-Szmirnov and Shapiro-Wilk tests. According to the analysis, the parameters showed a normal distribution. The homogeneity of the data was measured by the Levene test and, on it, homogeneity of the parameters was detected. Therefore, a variance analysis could be carried out and a one-way analysis of variance was used. The Bonferroni correction was used in multiple comparison tests.

The relative importance of different independent variables has been determined based on the Gini coefficient. This coefficient (also called the Gini indicator of impurity) indicates the probability of incorrect classification of a randomly selected entity. The Gini coefficient is similar to the entropy and suitable for measuring the degree of randomness or uncertainty. The decrease in the Gini coefficient after permutations of a given variable (expressed in %) indicates the importance of the present variable in prediction [33].

## 3. Results

### 3.1. Study Population

The basic parameters of the one hundred patients examined are shown in Table 1.

In the present population, 36 patients were examined as controls, while 32 participants were grouped as mild and 32 as moderately severe-severe OSA patients. As shown in Table 1, which summarises the gender, age and BMI categories of the groups, it can be concluded that most of the patients were men. The nutritional status was different in the various groups. Control patients are predominantly shown to be normal, patients in the mild OSA group to be overweight and obese and those in the moderately severe-severe group to be obese.

### 3.2. Basic Parameters of the OSA and Control Groups

The basic demographical, US and blood test parameters of the three groups are shown in Table 2.

Significant differences were observed between the OSA and the control groups in terms of age, body mass, BMI and neck, abdomen and hip circumferences, indicating significantly higher values in the OSA groups. When the US parameters of the SAT between the two groups were compared, most parameters (except the abdominal and the SAT thicknesses on the right side of the neck) were significantly higher in the OSA groups. In the case of blood test parameters, significantly lower HDL-cholesterol and higher triglyceride levels were observed in OSA patients.

### 3.3. Correlation between Anthropometric and US SAT Parameters and BMI and AHI 

The correspondence between anthropometric parameters and US SAT thickness values and AHI and BMI is presented in Table 3.

As shown in Table 3, BMI was correlated with almost anthropometric and US parameters. The most commonly expressed correlation was detected in the case of hip and abdominal circumferences and right and left-sided SAT thicknesses measured above the third intercostal spaces. The AHI was not significantly correlated with any parameters; the strongest correlation was observed in the case of the thickness of the right SAT above the third intercostal space and the thickness of the neck SAT.

### 3.4. Prediction of Oropharyngeal Obstruction

**Figure 2:**
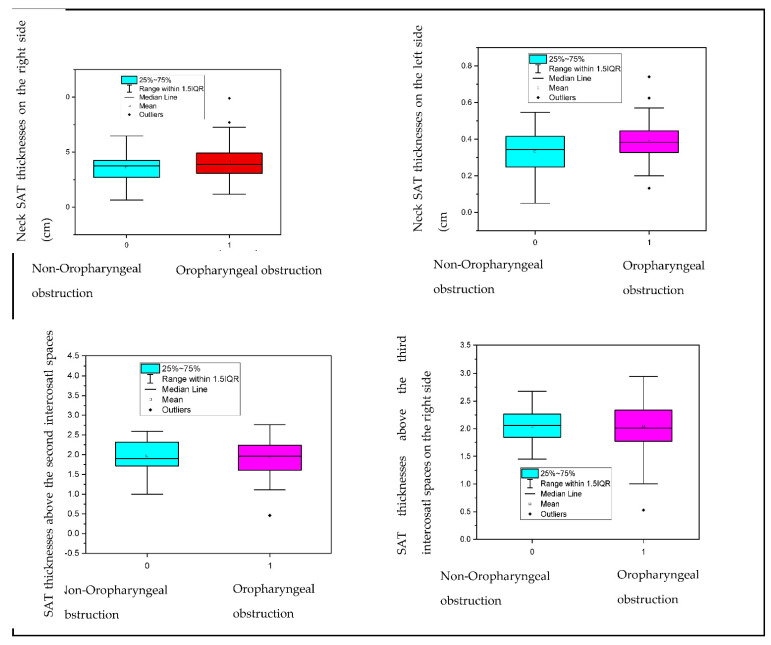

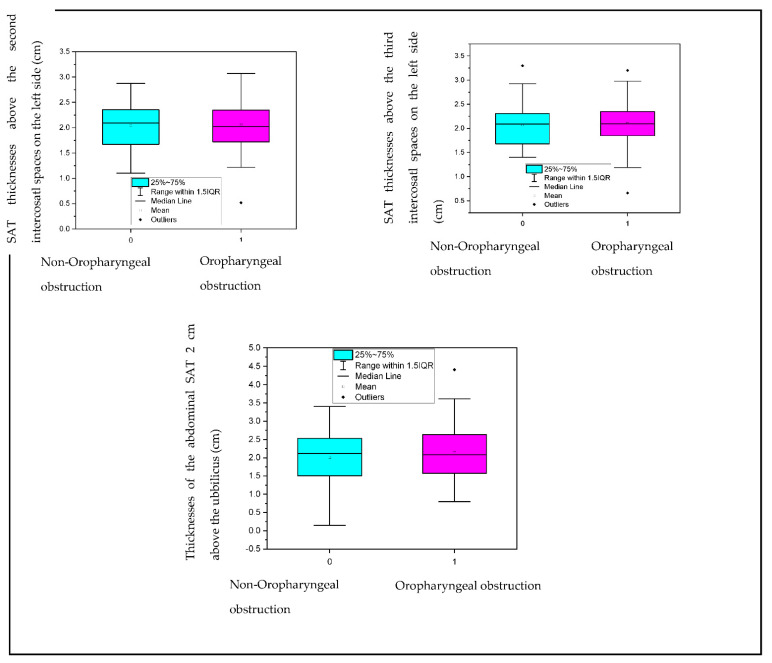
Correlation between SAT and oropharyngeal obstruction. IQR: interquartile range.

Prediction of oropharyngeal obstruction using artificial intelligence is demonstrated in Table 4 and the relative significance of each parameter is listed in Table 5. and Figure 2.

As Table 4 reveals, oropharyngeal obstruction was correctly predicted in 64%. In the other 16% of the cases, the algorithm identified a false oropharyngeal obstruction; furthermore, in 20%, it could not identify the existing obstruction.

As presented in Table 5, the algorithm determined BMI, body mass and abdominal circumference as the essential parameters for the identification of oropharyngeal obstruction, followed by the submental SAT thickness and the SAT thickness measured above the second intercostal space on the left side. The parameters mentioned above preceded such parameters as hip circumference and age. Moreover, the importance of neck circumference and gender was also of a small account contrasted to the SAT thicknesses based on the US. As presented in Figure 2, the SAT thicknesses measured in different locations were not significantly different between partial and total oropharyngeal obstruction.

### 3.5. Prediction of Tongue-Based Obstruction

Prediction of tongue-based obstruction using artificial intelligence is demonstrated in Table 6 and the relative significances of each parameter are listed in Table 7.

As can be concluded from Table 6, the algorithm was able to predict tongue-based obstruction in 72%. In the other 6%, a false positive classification was carried out and in 22%, the algorithm could not detect the existing tongue-based obstruction.

As can be seen from Table 7, the essential parameter in predicting tongue-based obstruction is height, followed by abdominal and submental SAT thicknesses. These parameters preceded others, such as age, BMI, sex, or neck circumferences.

### 3.6. Significance of Parameters in the Prognosis of OSA

According to the deep learning method, using Garson’s method, the relative significance of the anthropometric, blood test and US parameters was detected. The results are shown in Table 8.

In the prognosis of OSA, based on the deep learning method, the most critical parameter was the age of the examined patients, followed by sex and neck circumference. Using US parameters resulted in an average 34% increase in the effectiveness of the prediction model. In the case of the US parameters, the thickness of the SAT measured 2 cm above the umbilicus was detected as the most helpful parameter for the prognosis of OSA, while HDL-cholesterol levels were the essential laboratory test parameters. The thickness of the chest SAT, measured at the second intercostal spaces on the left and right sides, showed the same importance in predicting OSA as the BMI values. The importance of abdominal and hip SAT thicknesses was similar to triglyceride and cholesterol levels.

### 3.7. Prognosis of OSA Patients

The categorisation of non-OSA/ OSA patients, along with the different OSA categories, was based on anthropometric measurements, blood test parameters (i.e., cholesterol and triglyceride levels) and US parameters, using artificial intelligence. The results are shown in Table 9.

Based on anthropometric values, the parameters of the US and blood tests, OSA and its categories could be predicted with a precision of 97%. As shown in Table 9, the algorithm using artificial intelligence has failed to correctly prognosticate in only three cases of the control group, while in the mild and moderately severe-severe groups no failures were detected. Therefore, it can be concluded that, in addition to the precise categorisation of OSA and non-OSA patients, several OSA categories could be correctly predicted. With such precision, it can be expected that the algorithm has not ‘learned’, but only ‘mugged’ the correlations; thus, the correspondences have been mechanically used by it. To rule out this effect, the data were divided into ‘teaching’ and ‘testing’ groups, with a ratio of 75:25. Subsequently, the effectiveness of the prediction model was more than 90%, by running 100–100 analyses of the data.

## 4. Discussions

Although the pathogenesis of OSA is highly complex, obesity is one of the most critical risk factors for the disorder.

The present study aimed to analyse the correlation between US SAT thicknesses of the neck, chest and abdomen and the correlation between upper airway obstruction and OSA. Furthermore, the possible use of artificial intelligence in predicting OSA and upper airway obstruction, including SAT thicknesses, was also analysed. The improvement of the diagnostic work on OSA is of great importance given the high ratio of undiagnosed cases.

The prediction of OSA based on basic anthropometric parameters, SAT thicknesses of the neck, chest and abdomen regions was carried out using artificial intelligence, which is nowadays also used in medical science. According to its severity categories, OSA could be predicted with 97% precision using these parameters.

The most important parameters used by the algorithm were the thickness of the abdominal SAT, the thickness of the SAT above the second intercostal space on the right and left side, age, sex and neck circumference. The usefulness of abdominal SAT was detected as the same as in the case of neck circumference or gender. In the prognosis of OSA, the thickness of the chest SAT was of the same importance as BMI. Based on most clinical investigations, neck circumference, neck fold thickness, body mass and BMI were the most important predictors of OSA [34,35]. Moreover, it can also be emphasised that, in addition to gender, age and neck circumference, BMI and chest SAT thickness also have considerable effects on the pathogenesis of OSA; therefore, the examination of these parameters also contains much information for the diagnosis.

In general agreement with previous investigations, [36,37,38] in the current study, in the OSA group significantly higher values of neck circumference and SAT thickness were observed in the submental and submandibular region on the left side and both sides of the chest. However, none of the parameters mentioned above correlated with AHI; however, BMI was positively correlated with most anthropometric parameters and US SAT values.

Based on these correlations, the prognostic factors of OSA are essential in its prediction, although they are not correlated with the severity of OSA, i.e., based on AHI. Our results are similar to those of Ugur al., who have not observed a significant correlation between OSA severity (i.e., based on polysomnographic measurements) and US neck and abdominal SAT values [18]. Schäfer et al. have also not detected a correlation between the AHI index and the thickness of the neck SAT [39]. Oztura et al. found that the prediction of AHI is not influenced by BMI or neck circumference values [40]. Although Yagi and Plywaczesky et al. found a significant correlation between BMI, neck circumference and AHI detected [41,42], other investigations have also observed a strong correlation between OSA severity and neck circumference independently of visceral obesity [43,44]. Öǧretmenoǧlu et al. calculated body fat mass and body fat percentage as the parameter most correlated with AHI, based on bioelectric impedance analysis [45]. Liu et al. have found a higher thickness of abdominal and mesenteric adipose tissue in the OSA group, although the thickness of mesenteric adipose tissue showed a stronger correlation with AHI than the thickness of peritoneal and subcutaneous adipose tissues [46]. This result is consistent with that of Ma et al., who have not observed a strong correlation between abdominal SAT and OSA; however, visceral fat was determined as an OSA risk factor [47]. In the present study, the strongest correlation with BMI was detected between the SAT thicknesses of the chest and abdomen. Although neck SAT thickness might increase neck circumference, its correlation with BMI was moderate. Consistent with the results of the current study, Cielo et al. have also identified a correlation between adipose tissue in the neck and neck circumferences, although no correlation with OSA severity was detected [48].

The accumulation of adipose tissue near the upper airways results in a reduction in their diameters, an increasing tendency to extraluminal pressure and increased collapsibility during sleep. These mechanisms lead to upper airway obstructions [49].

The prediction of oropharyngeal obstruction using US parameters and anthropometric values was successful in 64%, which was determined using artificial intelligence. In addition to basic OSA risk factors, abdominal and chest SAT thicknesses had essential predictive values. The tongue-based obstruction could be predicted in 72% using the same parameters. Similarly to oropharyngeal obstruction, anthropometric parameters were crucial in OSA prediction; in addition, neck and abdominal SAT thicknesses were essential in this case. Therefore, it can be concluded that the thickness of the abdominal SAT corresponded to OSA and tongue-based obstruction, although it did not correspond to oropharyngeal obstruction. Submental and submandibular SAT was mainly correlated with oropharyngeal and tongue-based obstruction. This contradicts the results of Mortimore et al., who have stated that neck adipose tissue, independently of BMI values, significantly affects the occurrence of OSA; out of the anthropometric parameters, its correlation is the strongest with the disorder [50].

Interestingly, neck circumferences were identified as an essential factor in OSA prediction; however, they did not influence oropharyngeal or tongue-based obstruction, respectively.

SAT accumulation, as part of obesity, can result in OSA and upper airway obstruction in a highly complex way. OSA risk factors (e.g., sex, age and BMI) and SAT thicknesses of the neck, chest and abdomen can be helpful in the prediction of OSA with high precision. The oropharyngeal and tongue-based obstruction could be detected with a lower precision since many other factors can also be responsible for the obstructions, which were not examined in the present investigation.

The use of artificial intelligence has also occurred in sleep medicine in recent years, especially regarding OSA diagnosis. The main objective of different investigations was to predict OSA based on physiological effects during sleep, such as oxygen saturation, respiratory rate, or ECG parameters [51,52,53].

The use of self-administered questionnaires has some advantages in diagnosing OSA, e.g., they are easy to use, are not time-consuming and most give a reliable result. As previously reported, the prediction of OSA based on questionnaires by artificial intelligence showed a sensitivity of 80–81% and a specificity of 95–97% [54]. The NoSAS score, including age, sex, neck circumferences, snoring and obesity, presented a high sensitivity and positive predictive value in the diagnosis of OSA [55]. The STOP-BANG questionnaire showed high sensitivities and negative predictive values for diagnosing moderately severe OSA [56]. The SAS score can diagnose OSA with high sensitivity [57].

However, the prediction of OSA and upper airway obstruction based on SAT US and anthropometric parameters has not been previously investigated.

The significance of the present investigation is that using US in OSA diagnostics and obstruction localisation, an additional opportunity besides the standard procedures (i.e., drug-induced sleep endoscopy or polygraphy) is presented, which is vital due to the high account of undiagnosed cases. This can be especially significant in previously non-diagnosed cases of OSA when US examinations are indicated for other reasons (e.g., thyroid gland aberrations, salivary gland problems, abdominal pain or bilestones) or a US device is accessible for screening (e.g., general practitioner’s surgery, otorhinolaryngological or anaesthesiologic outpatient clinic). The applied algorithm can screen for OSA with high precision, which is essential, since the targeted diagnostic work-up for OSA can be started (home sleep test, polygraphy, PSG). Artificial intelligence can also help predict OSA and the location of the obstruction.

Our study has some limitations. Due to the relatively low number of investigated subjects, we were unable to divide OSA into more different groups. Examination of more subjects may provide facilities to carry out multivariable variance analyses. In the present study, visceral fat tissue was not investigated, although, according to the literature, it is also an essential factor in the pathogenesis of OSA.

## 5. Conclusions

The anatomical and non-anatomical characteristics of OSA are parts of the complexity of the disorder. All the anatomical parameters of OSA are measurable, resulting in a relatively significant number of variables. The analysis of non-linear correspondences with anthropometric and medical imaging parameters gives a possibility of a prediction based on learning. The importance of BMI in prediction was highlighted by its significant correlations with US parameters, which were not observed in the case of AHI. BMI was the essential parameter in the prediction of oropharyngeal obstruction and was also crucial in the prediction of OSA using artificial intelligence. Oropharyngeal obstruction could be detected in 64% and tongue-based obstruction in 72%. Abdominal circumference and submental adipose tissue were vital in oropharyngeal and tongue-based obstruction; however, they had a limited role in the prediction of OSA.

In addition to BMI, sex, age, neck circumference and some US parameters (i.e., thicknesses of abdominal SAT 2 cm above the umbilicus and SAT above the second intercostal space on the right side) also contained essential information concerning OSA prediction. Including these parameters, an appropriate OSA categorisation was performed in 97%. Consequently, artificial intelligence can effectively predict OSA and obstruction localisation. Given the high ratio of cases of undiagnosed OSA, improving its diagnosis is essential. In addition to the gold standard diagnostic methods SAT US and anthropometric measurements, analysis by artificial intelligence can be an alternative diagnostic method for OSA.

## Figures and Tables

**Table 1:** Distribution of the parameters of age, sex and BMI of the patients in the different groups.

Parameters	Categories	OSA Classification		
		Control (*n* = 36)	Mild OSA (*n* = 32)	Moderately Severe-Severe OSA (*n* = 32)
Gender	Men	21 (59%)	24 (75%)	29 (91%)
	Women	15 (41%)	8 (25%)	3 (9%)
Age	≤40 years	24 (67%)	9 (28%)	12 (37%)
	>40 years	12 (33%)	23 (72%)	20 (63%)
BMI	Normal	16 (44%)	2 (6%)	2 (6%)
	Overweight	12 (33%)	15 (47%)	10 (31%)
	Obese	8 (23%)	15 (47%)	20 (63%)

**Table 2:** Basic demographic, US and blood test results of the patients. The parameters show the mean ± SD values.

Parameters	Control Group *n* = 36 (A)	Mild OSA *n* = 32 (B)	Moderately Severe-Severe OSA *n* = 32 (C)	*p*-Value	Differences
Age (years)	38.42 ± 12.13	45.34 ± 11.17	43.16 ± 10.90	0.0003 ***	A-B
Weight (kg)	78.94 ± 13.15	93.03 ± 14.58	101.97 ± 17.21	<0.001 ***	A-B;A-C;B-C
BMI (kg/m^2^)	26.10 ± 3.83	30.73 ± 5.81	31.49 ± 4.77	<0.001 ***	A-B; A-C
Hip circumference (cm)	100.49 ± 11.97	106.28 ± 10.46	111.15 ± 10.96	0.001 ***	A-C
Abdominal circumference (cm)	94.73 ± 12.70	104.97 ± 11.68	111.01 ± 12.51	<0.001 ***	A-B;A-C
Neck circumference (cm)	37.95 ± 4.12	40.69 ± 3.42	42.73 ± 3.33	<0.001 ***	A-B;A-C;B-C
Submental SAT thickness (cm)	0.63 ± 0.31	0.66 ± 0.24	0.86 ± 0.32	0.001 ***	A-C;B-C
Neck SAT thickness on the right side (cm)	0.34 ± 0.13	0.40 ± 0.13	0.44 ± 0.17	0.127	No significant difference
Neck SAT thickness on the left side (cm)	0.33 ± 0.11	0.38 ± 0.13	0.40 ± 0.11	0.03 **	A-C
SAT thickness above the second intercostal space on the right side (cm)	0.52 ± 0.24	0.73 ± 0.37	0.70 ± 0.32	0.013 **	A-B;A-C
SAT thickness above the third intercostal space on the right side (cm)	0.72 ± 0.31	0.93 ± 0.38	0.90 ± 0.33	0.092 *	A-B
SAT thickness above the second intercostal space on the left side (cm)	0.58 ± 0.30	0.79 ± 0.43	0.84 ± 0.37	0.01 **	A-B;A-C
SAT thickness above the third intercostal space on the left side (cm)	0.75 ± 0.34	1.02 ± 0.40	1.02 ± 0.36	0.003 ***	A-B;A-C
Thickness of abdominal SAT 2 cm above the umbilicus (cm)	1.95 ± 0.72	2.21 ± 0.85	2.14 ± 0.69	0.328	No significant difference
Total cholesterol (mmol/L)	5.59 ± 1.15	5.90 ± 1.17	5.47 ± 1.01	0.279	No significant difference
HDL-cholesterol (mmol/L)	1.31 ± 0.29	1.22 ± 0.32	1.13 ± 0.17	0.024 **	A-C
LDL-cholesterol (mmol/L)	3.58 ± 0.81	3.89 ± 0.82	3.70 ± 0.80	0.282	No significant difference
Triglycerides (mmol/L)	1.82 ± 1.33	2.58 ± 1.80	2.04 ± 1.17	0.097 *	A-B

*** indicates the significant difference at *p* < 0.01 level, while ** the significant difference at *p* < 0.05 level and * the significant difference at *p* < 0.1.

**Table 3:** Correlation between anthropometric and US SAT parameters and BMI and AHI. The parameters show the correlation coefficients.

Parameters	Correlation with	
	BMI	AHI
	r value	r value
Age (year)	0.015	0.066
Hip circumference (cm)	0.793 **	0.011
Abdominal circumference (cm)	0.872 **	−0.014
Neck circumference (cm)	0.357 **	−0.035
Submental SAT thickness (cm)	0.392 **	0.032
Neck SAT thickness on the right side (cm)	0.399 **	0.154
Neck SAT thickness on the left side (cm)	0.412 **	−0.042
SAT thickness above the second intercostal space on the right side (cm)	0.510 **	0.001
SAT thickness above the third intercostal space on the right side (cm)	0.571 **	0.105
SAT thickness above the second intercostal space on the left side (cm)	0.544 **	0.037
SAT thickness above the third intercostal space on the left side (cm)	0.572 **	0.046
Abdominal SAT thickness 2 cm above the umbilicus (cm)	0.567 **	−0.057

*** indicates the significant difference at *p* < 0.01 level, while ** the significant difference at *p* < 0.05 level and * the significant difference at *p* < 0.1.

**Table 4:** The prediction of oropharyngeal obstruction using US SAT thickness parameters by artificial intelligence. In the table, the percentage values are visualised.

		Reference	
		Non-Oropharyngeal Obstruction	Oropharyngeal Obstruction
Prediction	Non-Oropharyngeal obstruction	14	20
	Oropharyngeal obstruction	16	50

**Table 5:** Relative significances of the US and anthropometric parameters in the prediction of oropharyngeal obstruction, using artificial intelligence.

Indicators	Mean Decrease in Gini (%)
BMI	7.28
Weight	3.67
Abdominal circumference	3.20
Submental SAT thickness	2.98
SAT thickness above the second intercostal spaces on the left side	2.97
SAT thickness on the left side	2.90
Hip circumference	2.90
Age	2.85
SAT thickness above the third intercostal spaces on the left side	2.35
SAT thickness above the second intercostal spaces on the right side	2.35
Abdominal SAT thickness 2 cm above the umbilicus	2.31
SAT thickness above the third intercostal space on the right side	2.23
Neck SAT thickness on the right side	2.17
Height	2.06
Neck circumference	1.82
Gender	0.45

**Table 6:** The prediction of tongue-based obstruction using US SAT thickness parameters by artificial intelligence. In the table, the percentage values are visualised.

		Reference	
		Non- Tongue- Based Obstruction	Tongue-Based Obstruction
Prediction	Non-Tongue- based obstruction	7	22
	Tongue-based obstruction	6	65

**Table 7:** Relative significance of the US and anthropometric parameters in the prediction of tongue-based obstruction, using artificial intelligence.

Parameters	Mean Decrease in Gini (%)
Height	4.67
Abdominal SAT thickness 2 cm above the umbilicus	3.57
Submental SAT thickness	3.49
Age	3.39
Weight	2.84
SAT thickness above the second intercostal space on the left side	2.78
SAT thickness above the third intercostal space on the left side	2.63
SAT thickness on the left side	2.51
Abdominal circumference	2.51
Neck SAT thickness on the right side	2.37
BMI	2.34
SAT thickness above the third intercostal space on the right side	2.28
Neck circumference	1.97
SAT thickness above the second intercostal space on the right side	1.83
Hip circumference	1.59
Gender	0.27

**Table 8:** Relative significance of anthropometric, blood test and US parameters in the prognostication of OSA.

Parameters	Importance (%)
Age	12.30
Gender	7.80
Neck circumference	7.56
Thickness of abdominal SAT 2 cm above the umbilicus	7.50
Thickness of SAT above the second intercostal space on the right side	6.78
BMI	6.75
HDL-cholesterol	6.10
Thickness of SAT above the second intercostal space on the left side	5.73
LDL-cholesterol	5.58
Height	4.05
Thickness of neck SAT on the left side	3.85
Triglycerides	3.65
Abdominal circumference	3.60
Hip circumference	3.05
Total cholesterol	3.00
Thickness of SAT above the third intercostal space on the left side	2.95
Thickness of neck SAT on the right side	2.80
Thickness of SAT above the third intercostal space on the right side	2.80
Body mass	2.15
Thickness of submental SAT	2.00

**Table 9:** Results of the categorisation of OSA/non-OSA groups using artificial intelligence, based on anthropometric and laboratory test parameters and US measurements.

Estimated	OSA Categories		
	Control (*n* = 36)	Mild OSA (*n* = 32)	Moderately Severe-Severe OSA (*n* = 32)
Control	33		
Mild OSA	3	32	
Moderately severe-severe OSA			32

## Data Availability

Data supporting reported results can be provided upon reasonable request.

## References

[1] Antonaglia C., Passuti G. (2021). Obstructive sleep apnea syndrome in non-obese patients. Sleep Breath.

[2] McCloy K., Duce B., Swarnkar V., Hukins C., Abeyratne U. (2021). Polysomnographic risk factors for vigilance-related cognitive decline and obstructive sleep apnea. Sleep Breath.

[3] Kabak M., Akbudak M. (2021). Obstructive sleep apnea syndrome as a potential cause of sexual dysfunction in women. Sleep Breath.

[4] Popkin B.M., Adair L.S., Ng S.W. (2012). Global nutrition transition and the pandemic of obesity in developing countries. Nutr. Rev..

[5] Prentice A., Jebb S. (2004). Energy intake/physical activity interactions in the homeostasis of body weight regulation. Nutr. Rev..

[6] Ng M., Fleming T., Robinson M., Thomson B., Graetz N., Margono C., Mullany E.C., Biryukov S., Abbafati C., Abera S.F. (2014). Global, regional, and national prevalence of overweight and obesity in children and adults during 1980–2013: A systematic analysis for the Global Burden of Disease Study 2013. Lancet.

[7] Ryan S., Arnaud C., Fitzpatrick S.F., Gaucher J., Tamisier R., Pépin J.L. (2019). Adipose tissue as a key player in obstructive sleep apnoea. Eur. Respir. Rev..

[8] Feinstein A.R. (1977). Clinical biostatistics. Clin. Pharmacol. Ther..

[9] Singh S., Makharia A., Chakrabarti A. (2019). Modelling of Causal Relations in Human Pathophysiology for Medical Education and Design Inspiration. Research into Design for a Connected World.

[10] Bujang M.A., Sa’at N., Sidik T.M.I.T.A.B., Joo L.C. (2018). Sample Size Guidelines for Logistic Regression from Observational Studies with Large Population: Emphasis on the Accuracy Between Statistics and Parameters Based on Real Life Clinical Data. Malays. J. Med. Sci..

[11] Park L.Q., Gross A.L., McLaren D.G., Pa J., Johnson J.K., Mitchell M., Manly J.J. (2012). Confirmatory factor analysis of the ADNI neuropsychological battery. Brain Imaging Behav..

[12] Koran J. (2020). Indicators per factor in confirmatory factor analysis: More is not always better. Struct. Equ. Model. A Multidiscip. J..

[13] Kirby S.D., Danter W., George C.F., Francovic T., Ferguson K.A., Eng P., Ruby R.R. (1999). Neural network prediction of obstructive sleep apnea from clinical criteria. Chest.

[14] El-Solh A.A., Mador M.J., Ten-Brock E., Shucard D.W., Abul-Khoudoud M., Grant B.J. (1999). Validity of neural network in sleep apnea. Sleep.

[15] Karamanli H., Yalcinoz T., Yalcinoz M.A., Yalcinoz T. (2016). A prediction model based on artificial neural networks for the diagnosis of obstructive sleep apnea. Sleep Breath.

[16] Berry R.B., Budhiraja R., Gottlieb D.J., Gozal D., Iber C., Kapur V.K., Marcus C.L., Mehra R., Parthasarathy S., Quan S.F. (2012). Rules for scoring respiratory events in sleep: Update of the 2007 AASM manual for the scoring of sleep and associated events: Deliberations of the sleep apnea definitions task force of the American Academy of Sleep Medicine. J. Clin. Sleep Med..

[17] Shu C.C., Lee P., Lin J.W., Huang C.T., Chang Y.C., Yu C.J., Wang H.C. (2013). The use of sub-mental ultrasonography for identifying patients with severe obstructive sleep apnea. PLoS ONE.

[18] Ugur K.S., Ark N., Kurtaran H., Kizilbulut G., Cakir B., Ozol D., Gunduz M. (2011). Subcutaneous fat tissue thickness of the anterior neck and umbilicus in patients with obstructive sleep apnea. Otolaryngol. Head Neck Surg..

[19] Stanley J.J. (2020). Drug-induced sleep endoscopy: Techniques, interpretation and implications. Curr. Opin. Pulm. Med..

[20] Ziegel E.R. (2004). Statistics and chemometrics for analytical chemistry. Technometrics.

[21] Hastie T., Tibshirani R., Leisch F., Hornik K., Ripley B. (2022). mda: Mixture and Flexible Discriminant Analysis.

[22] Schmidhuber J. (2015). Deep learning in neural networks: An overview. Neural Netw..

[23] Zhang Y., Mo Q., Xue L., Luo J. (2021). Evaluation of deep learning approaches for modeling transcription factor sequence specificity. Genomics.

[24] Lee G., Fujita H. (2020). Deep Learning in Medical Image Analysis: Challenges and Applications.

[25] Zhang Z., Rousson V., Lee W., Ferdynus C., Chen M., Qian X. (2018). AME Big-Data Clinical Trial Collaborative Group. Decision curve analysis: A technical note. Ann. Transl. Med..

[26] Prajwala T. (2015). A comparative study on decision tree and random forest using R tool. Int. J. Adv. Res. Comput. Commun. Eng..

[27] Giorgi F.M., Ceraolo C., Mercatelli D. (2022). The R Language: An Engine for Bioinformatics and Data Science. Life.

[28] Jungbauer F., Gerhards C., Thiaucourt M., Behnes M., Rotter N., Schell A., Haselmann V., Neumaier M., Kittel M. (2022). Anosmia Testing as Early Detection of SARS-CoV-2 Positivity; A Prospective Study under Screening Conditions. Life.

[29] Liu C.-M., Ta V.-D., Le N.Q.K., Tadesse D.A., Shi C. (2022). Deep Neural Network Framework Based on Word Embedding for Protein Glutarylation Sites Prediction. Life.

[30] Rong X., Rong M.X. (2014). Package ‘Deepnet’. https://cran.microsoft.com/snapshot/2015-01-15/web/packages/deepnet/deepnet.pdf.

[31] Kuhn M. (2008). Building predictive models in R using the caret package. J. Stat. Softw..

[32] Beck M.W. (2018). NeuralNetTools: Visualization and analysis tools for neural networks. J. Stat. Softw..

[33] Zhang Y., Wen J., Yang G., He Z., Wang J. (2019). Path loss prediction based on machine learning: Principle, method, and data expansion. Appl. Sci..

[34] Davies R. (1990). The relationship between neck circumference, radiographic pharyngeal anatomy, and the obstructive sleep apnoea syndrome. Eur. Respir. J..

[35] Cizza G., de Jonge L., Piaggi P., Mattingly M., Zhao X., Lucassen E., Rother K.I., Sumner A.E., Csako G. (2014). Neck circumference is a predictor of metabolic syndrome and obstructive sleep apnea in short-sleeping obese men and women. Metab. Syndr. Relat. Disord..

[36] Ahbab S., Ataoğlu H.E., Tuna M., Karasulu L., Çetin F., Temiz L.Ü., Yenigün M. (2013). Neck circumference, metabolic syndrome and obstructive sleep apnea syndrome; evaluation of possible linkage. Med. Sci. Monit..

[37] Soylu A.C., Levent E., Sarıman N., Yurtlu Ş., Alparslan S., Saygı A. (2012). Obstructive sleep apnea syndrome and anthropometric obesity indexes. Sleep Breath.

[38] Hoffstein V., Mateika S. (1992). Differences in abdominal and neck circumferences in patients with and without obstructive sleep apnoea. Eur. Respir. J..

[39] Scha H., Pauleit D., Sudhop T., Gouni-Berthold I., Ewig S., Berthold H.K. (2002). Body fat distribution, serum leptin, and cardiovascular risk factors in men with obstructive sleep apnea. Chest.

[40] Oztura I., AKDOĞAN O., YENER G.G., BAKLAN B. (2013). Influence of Gender, Obesity and Neck Circumference on Sleep-Disordered Breathing in A Sleep Referral Center. J. Neur. Sci..

[41] Yagi H., Nakata S., Tsuge H., Yasuma F., Noda A., Morinaga M., Tagaya M., Nakashima T. (2009). Morphological examination of upper airway in obstructive sleep apnea. Auris Nasus Larynx.

[42] Pływaczewski R., Bieleń P., Bednarek M., Jonczak L., Górecka D., Śliwiński P. (2008). Influence of neck circumference and body mass index on obstructive sleep apnoea severity in males. Pneumonol. Alergol. Pol..

[43] Kawaguchi Y., Fukumoto S., Inaba M., Koyama H., Shoji T., Shoji S., Nishizawa Y. (2011). Different impacts of neck circumference and visceral obesity on the severity of obstructive sleep apnea syndrome. Obesity.

[44] Yildirim Y., Yilmaz S., Güven M., Kılınç F., Kara A.V., Yilmaz Z., Kırbaş G., Tuzcu A.K., Aydın F.Y. (2015). Evaluation of anthropometric and metabolic parameters in obstructive sleep apnea. Pulm. Med..

[45] Öğretmenoğlu O., Süslü A.E., Yücel Ö.T., Önerci T.M., Şahin A. (2005). Body fat composition: A predictive factor for obstructive sleep apnea. Laryngoscope.

[46] Liu K.H., Chu W.C.W., To K.W., Ko F.W.S., Ng S.S.S., Ngai J.C.L., Chan J.W.S., Ahuja A.T., Hui D.S.C. (2014). Mesenteric fat thickness is associated with increased risk of obstructive sleep apnoea. Respirology.

[47] Ma B., Li Y., Wang X., Du L., Wang S., Ma H., Zhou D., Usman T., Lu L., Qu S. (2022). Association Between Abdominal Adipose Tissue Distribution and Obstructive Sleep Apnea in Chinese Obese Patients. Front. Endocrinol..

[48] Cielo C.M., Keenan B.T., Wiemken A., Tapia I.E., Kelly A., Schwab R.J. (2021). Neck fat and obstructive sleep apnea in obese adolescents. Sleep.

[49] Kairaitis K., Howitt L., Wheatley J.R., Amis T.C. (2009). Mass loading of the upper airway extraluminal tissue space in rabbits: Effects on tissue pressure and pharyngeal airway lumen geometry. J. Appl. Physiol..

[50] Mortimore I.L., Marshall I., Wraith P.K., Sellar R.J., Douglas N.J. (1998). Neck and total body fat deposition in nonobese and obese patients with sleep apnea compared with that in control subjects. Am. J. Respir. Crit. Care Med..

[51] Álvarez D., Cerezo-Hernández A., Crespo A., Gutiérrez-Tobal G.C., Vaquerizo-Villar F., Barroso-García V., Moreno F., Arroyo C.A., Ruiz T., Hornero R. (2020). A machine learning-based test for adult sleep apnoea screening at home using oximetry and airflow. Sci. Rep..

[52] Papini G.B., Fonseca P., van Gilst M.M., van Dijk J.P., Pevernagie D.A., Bergmans J.W., Vullings R., Overeem S. (2019). Estimation of the apnea-hypopnea index in a heterogeneous sleep-disordered population using optimised cardiovascular features. Sci. Rep..

[53] Behar J.A., Palmius N., Li Q., Garbuio S., Rizzatti F.P., Bittencourt L., Tufik S., Clifford G.D. (2019). Feasibility of single channel oximetry for mass screening of obstructive sleep apnea. Clin. Med..

[54] Sun L.M., Chiu H.-W., Chuang C.Y., Liu L. (2011). A prediction model based on an artificial intelligence system for moderate to severe obstructive sleep apnea. Sleep Breath.

[55] Costa J.C., Rebelo-Marques A., Machado J.N., Gama J., Santos C., Teixeira F., Moita J. (2019). Validation of NoSAS (Neck, Obesity, Snoring, Age, Sex) score as a screening tool for obstructive sleep apnea: Analysis in a sleep clinic. Pulmonology.

[56] Chung F., Abdullah H.R., Liao P. (2016). STOP-Bang questionnaire: A practical approach to screen for obstructive sleep apnea. Chest.

[57] Topîrceanu A., Udrescu M., Udrescu L., Ardelean C., Dan R., Reisz D., Mihaicuta S. (2018). SAS score: Targeting high-specificity for efficient population-wide monitoring of obstructive sleep apnea. PLoS ONE.

